# Coronary Bifurcation PCI—Part II: Advanced Considerations

**DOI:** 10.3390/jcdd12110439

**Published:** 2025-11-06

**Authors:** Rongras Damrongwatanasuk, Sara Pollanen, Ju Young Bae, Jason Wen, Michael G. Nanna, Abdulla A. Damluji, Mamas A Mamas, Elias B. Hanna, Jiun-Ruey Hu

**Affiliations:** 1 Division of Cardiovascular Medicine, Department of Internal Medicine, University of Louisville, Kentucky, KY 40202, USA; 2 Temerty Medicine, University of Toronto, Toronto, ON M5S 1A1, Canada; 3 Division of Cardiovascular Medicine, Weill Cornell Medicine, New York Presbyterian Hospital, New York, NY 10032, USA; 4 Department of Cardiology, Smidt Heart Institute, Cedars-Sinai Medical Center, Los Angeles, CA 90048, USA; 5 Department of Medicine, Section of Cardiovascular Medicine, Yale New Haven Hospital, New Haven, CT 06510, USA; 6 Cardiovascular Center on Aging, Department of Cardiovascular Medicine, Cleveland Clinic Foundation, Cleveland, OH 44195, USA; 7 Keele Cardiovascular Research Group, Keele University, Newcastle ST5 5BG, UK; 8 Cardiology Department, Lebanese American University, Beirut 1102, Lebanon

**Keywords:** primary coronary intervention, bifurcation lesions, bifurcation stenting, coronary artery disease

## Abstract

Performance of percutaneous coronary intervention (PCI) for bifurcation lesions involves complex decision-making informed by anatomic, hemodynamic, technical, and clinical factors. Building on the procedural foundations discussed in the companion paper in this two-part series, this second paper focuses on advanced considerations in bifurcation PCI. Factors associated with side branch (SB) compromise are discussed, including bifurcation angle and distribution of plaque location, along with strategies for SB protection and SB rewiring. Outcomes of landmark randomized controlled trials of provisional versus two-stent approaches, as well as specific two-stent techniques, are summarized. Based on these factors, an algorithmic approach for bifurcation PCI is outlined. Lastly, the use of drug-coated balloons, dedicated bifurcation stents, bioresorbable vascular scaffolds, bioadaptor stents, physiologic testing, intravascular imaging, and other emerging innovations are explored to provide a perspective on the future of bifurcation PCI.

## 1. Introduction

Coronary bifurcation lesions continue to be a challenge in percutaneous coronary intervention (PCI) due to the inherent tradeoffs between side branch (SB) protection, stent optimization, and long-term vessel patency. While technique selection is often guided by lesion anatomy and operator familiarity, there is growing recognition of the importance of individualized, physiology-guided, and data-driven approaches. The European Bifurcation Club recommends a provisional stenting strategy as the standard approach for many bifurcation lesions, while in complex bifurcation lesions upfront two-stent techniques may be used [1]. In the first part of this two-part review, “Fundamentals of Coronary Bifurcation PCI”, we reviewed major bifurcation lesion classification systems and procedural strategies, with a special focus on the visualization of step-by-step techniques for the practicing clinician [2]. In the present paper, “Advanced Considerations in Coronary Bifurcation PCI”, we review considerations for decision-making regarding SBs, including factors that predict SB compromise, situations that call for a two-stent approach upfront, and the role of physiologic testing. On a practical level, we review techniques for active and passive protection of the SB, techniques for maintaining and rewiring the SB, and approaches to guidewire entrapment. With evidence-based practice in mind, we review randomized trial data associated with different intervention techniques. Integrating these considerations, we provide an algorithmic approach to selecting the optimal technique for a given patient. Finally, we review emerging developments such as the role of drug-coated balloons, dedicated bifurcation stents, bioresorbable scaffolds, and bioadaptor stents.

## 2. SB Considerations

### 2.1. Factors Predicting SB Compromise

**Figure 1:**
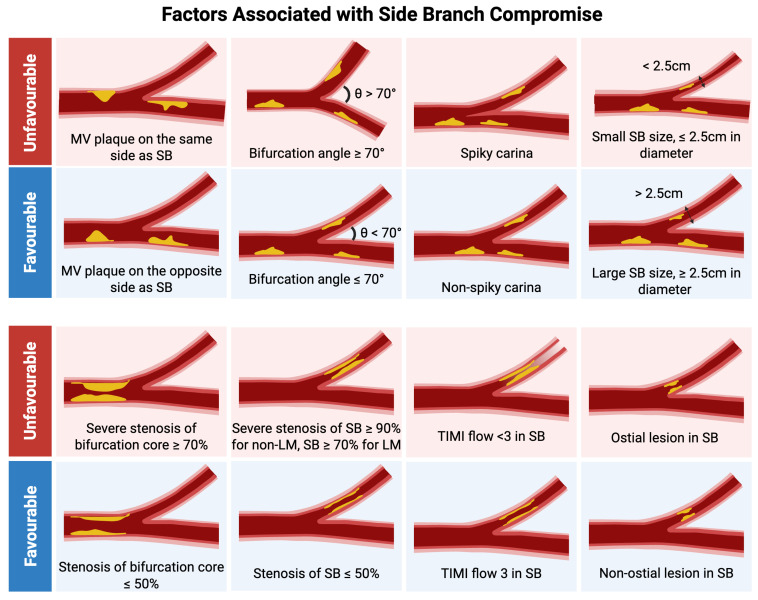
Factors associated with side branch (SB) compromise. Original illustration.

There are several anatomical and procedural risk factors that predict when the SB may be at risk of compromise (Figure 1) [3,4,5]:

*Plaque on the same side of the SB*: this refers to atherosclerotic plaque located on the side of the main vessel (MV) directly opening to the origin of the SB, in contrast with atherosclerotic plaque located on the side of the MV opposite to the origin of the SB. During MV stent deployment, this plaque is more likely to shift into the SB ostium, increasing the risk of occlusion.

*Unfavorable bifurcation angle ≥ 70°*: A wide angle (greater than 70°) between the MV and the SB makes it more difficult to access and protect the SB. Additionally, it alters the flow dynamics, making the SB more susceptible to plaque shift and hemodynamic compromise. A more gradual angle (0–70°) generally allows easier access to the SB and reduces the risk of compromise.

*Spiky carina*: A sharply pointed carina (central ridge between MV and SB) identified on imaging (e.g., optical coherence tomography [OCT] or intravascular ultrasound [IVUS]) indicates unstable geometry. MV stenting can displace this structure into the SB, contributing to compromise or occlusion.

*Small SB size*: SBs with a smaller diameter < 2.5 cm are more vulnerable to compromise. A large main vessel (MV) relative to the SB (defined as a high ratio MV diameter to SB diameter of ≥2) suggests a significant size mismatch. During MV stent expansion, this can cause disproportionate plaque shift into the smaller SB, which has less margin for handling plaque shift without resulting in obstruction.

*Severe % diameter stenosis of bifurcation core ≥ 70%*: The bifurcation core is the central area encompassing the MV just before and after the SB takeoff. A high-grade stenosis of ≥70% suggests that the large plaque burden may easily shift or “snowplow” into the SB during stenting.

*Severe % diameter stenosis at SB ≥ 90% for non-left main (LM) lesions and ≥70% for LM lesions*: High-grade stenosis at the SB ostium or shaft before intervention increases the chance of total occlusion when additional plaque is displaced during MV stenting.

*Reduced TIMI flow at the SB*: TIMI (Thrombolysis in Myocardial Infarction) flow grade < 3 in the SB at baseline indicates impaired perfusion, often due to existing lesion severity or microvascular dysfunction. Lower flow suggests the SB is more vulnerable and less likely to tolerate any additional obstruction.

*Ostial lesion location on the SB*: if the lesion is located proximally or ostially on the SB (closer to the bifurcation), the SB is more likely to be compromised.

The RESOLVE (Risk prEdiction of SB OccLusion in coronary bifurcation interVEntion) score is a validated angiographic risk stratification tool, which can help identify patients at risk of SB occlusion during bifurcation intervention [6]. The QCA-based RESOLVE score system contains six independent risk factors for SB occlusion: two visual estimation predictors (plaque distribution and MV TIMI flow grade before stenting), and four quantitative coronary angiography (QCA) analysis predictors (pre-procedural diameter stenosis of bifurcation core, bifurcation angle, diameter ratio between MV/SB, and diameter stenosis of SB before MV stenting). The RESOLVE score ranges from 0 to 43. Rates of SB occlusion are 0% in the low-risk group (score 0–2), 3.8% in the intermediate-risk group (score 3–9), and 19.8% in the high-risk group (score 10–42) [7].

Although QCA provides a more objective determination of the extent and severity of coronary artery disease, it is not available for real time use in most cardiac catheterization laboratories and thus is rarely used prospectively in clinical settings. As a result, the inclusion of QCA data within the QCA-based RESOLVE score limits its ability to be used at the time of bifurcation intervention. To address this limitation, a non-QCA score was developed that relies solely on visual angiographic findings, the V-RESOLVE score [6,7].

### 2.2. Plaque Shift and Carina Shift

Plaque shift occurs when the atherosclerotic plaque (the fatty deposit or lesion) in the MV is displaced during the intervention. When a balloon is inflated or a stent is deployed in the MV, the pressure exerted on the plaque can cause it to shift into the SB, obstructing the flow in the SB or narrowing its opening. This is particularly problematic when the plaque is displaced towards the ostium (the opening) of the SB, resulting in a reduction in flow to the SB. The SB may become stenosed or even occluded, depending on the degree of plaque displacement. A larger, more complex plaque in the MV or a sharp angulation at the bifurcation increases the likelihood of plaque shift [8].

Carina shift refers to the movement or displacement of the carina, which is the ridge or dividing line between the MV and the SB at the bifurcation point. The carina is the anatomical feature where the MV branches off into the SB. When a balloon is inflated or a stent is deployed, it can cause the carina to move upward. This is particularly true if excessive force is applied during the procedure or if the bifurcation angle is steep. If the carina moves toward the SB, the angle between the SB and the MV may become sharper, making it more difficult to access or stent the SB [8].

### 2.3. Situations That Call for a Two-Stent Approach Upfront

When should you consider a two-stent approach from the start, instead of provisional stenting? While provisional stenting (single stent in the MV) is the preferred method for most bifurcation lesions, a two-stent strategy (stenting both the MV and the SB) is recommended if a bifurcation lesion is a complex bifurcation lesion. The DEFINITION criteria for complex coronary bifurcation lesions, developed from the 2014 DEFINITION study (Definitions and impact of complEx biFurcation lesIons on clinical outcomes after percutaNeous coronary IntervenTIOn using drug-eluting steNts), consists of two major and six minor angiographic criteria [9,10,11,12]. A bifurcation lesion is defined as complex if at least one major criterion and two or more minor criteria are present (Table 1) [10]. Building upon the DEFINITION criteria, the DEFINITION II trial compared outcomes of two-stent techniques versus provisional stenting in patients with complex bifurcation lesions [13]. For lesions under the DEFINITION criteria, a two-stent approach may offer better clinical outcomes, with the two-stent group exhibiting lower rates of target lesion failure (6.1% compared to 11.4%, respectively, 95% CI 0.30–0.90; *p* = 0.019) [13]. This was also shown in the DK-CRUSH V trial of LM bifurcation stenting, wherein the subgroup of patients meeting the DEFINITION criteria derived a significant benefit from a two-stent strategy compared to provisional stenting, whereas patients not meeting those criteria did not [14].

### 2.4. Role of Physiologic Testing for SB Protection

There remains insufficient evidence to support the role of Fractional Flow Reserve (FFR) and Instantaneous Wave-Free Ratio (iFR) for guiding whether to protect the SB in coronary bifurcation lesions. Both DEFINE-FLAIR and SWEDEHEART studied iFR and FFR in general, not specifically their value in bifurcation disease [15,16]. The EBC and its counterparts note that it is not possible to use pre-intervention IFR/FFR to determine the bifurcation treatment strategy (one stent or upfront double stenting) or to predict the functional significance of a SB, because the SB is subject to the nature of MV stenosis and the dynamic geometric changes that occur during PCI from plaque shift and carina shift, factors which are not accounted for in physiologic testing of the SB pre-intervention [17].

However, FFR may have a role in the assessment of the SB post-MB (main branch) stenting in the provisional strategy. In one study, among 73 non-LM bifurcations with short SB lesions (<10 mm) whose stenoses exceeded 75% after MV stenting, only 27% were functionally significant according to FFR. Even in the case of SBs with vessel diameters of ≥2.5 mm, FFR was significant in only 38% of the cases. FFR was rarely significant in the case of SB stenosis < 90%. Even for SB stenosis > 90%, FFR was only significant around half the time. Thus, most stenoses of jailed SBs are not functionally significant, even when severe, and may not need rewiring and balloon dilatation until they are >75–90% obstructive. This may be related to the fact that SB narrowing after MB stenting is frequently secondary to carina shift, which distorts the ostium without necessarily narrowing it three-dimensionally [18].

## 3. SB Protection, Wiring, and Rewiring

### 3.1. Techniques for Passive and Active SB Protection

**Figure 2:**
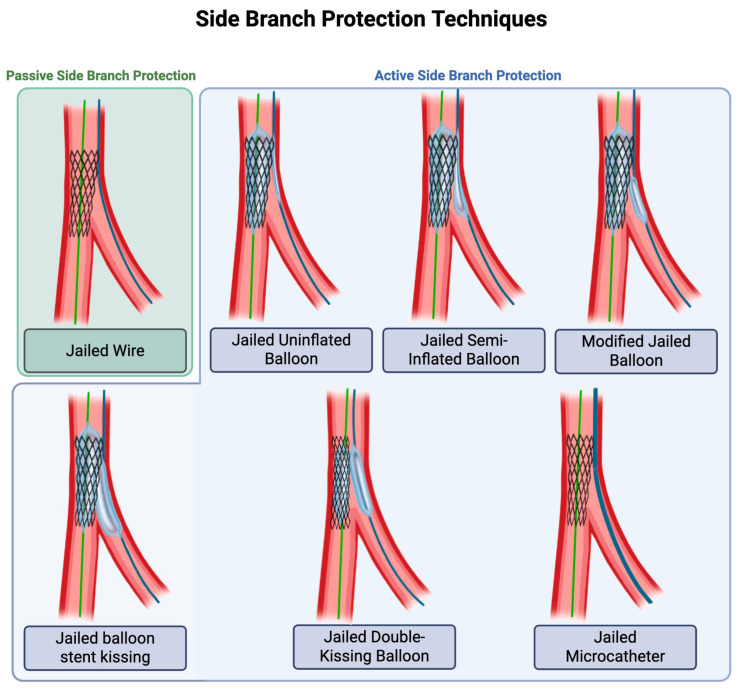
Active and passive protection strategies to mitigate the risk of side branch (SB) occlusion.

In addition to the widely used jailed wire technique, which passively preserves access to the SB during MV stenting, several active SB protection strategies have been developed to further mitigate the risk of SB occlusion (Figure 2) [19]. The jailed uninflated balloon technique, first described by Burzotta et al., involves advancing a small, uninflated balloon into the SB, which is then jailed during MV stent deployment [20]. This uninflated balloon helps reduce mechanical plaque shift into the SB, in addition to preserving access to the SB. If the SB becomes occluded, the jailed balloon can be inflated to restore flow. If the SB does not become occluded, the SB balloon can be withdrawn, followed by POT.

In the jailed semi-inflated balloon technique, the SB balloon is inflated at low pressure (3–4 atm), while the MV stent is deployed at nominal pressure [21]. The proximal segment of the SB balloon is compressed against the vessel wall as the MV stent inflates, while the distal segment of the SB balloon becomes over-inflated and completely occupies the SB ostium, thus preventing plaque shift. If the SB does not become occluded, the SB balloon can be withdrawn, followed by POT. Please note that in the jailed uninflated balloon technique and the jailed semi-inflated balloon technique, the proximal marker of the SB balloon is positioned such that it is proximal to the proximal edge of the MB stent, which is not the case in the subsequent techniques.

In the modified jailed balloon technique, the SB balloon is placed in the SB with a 1 mm protrusion into the MB [22,23], whereas in the jailed balloon stent kissing technique and the jailed double-kissing balloon technique, the SB balloon is placed proximally in the MB, although care is taken to ensure it does not protrude proximally to the MB stent [24,25]. In the jailed balloon stent kissing technique, the SB balloon is inflated at low pressure (6–8 atm), while the MV stent is deployed at nominal pressure. After the jailed SB balloon is deflated and removed, with the wire left in the SB, the MV stent balloon is fully inflated, to correct any stent deformation that was present during the initial inflation. In the jailed double-kissing balloon outside the stent technique, the SB balloon is used for two inflations: during the first kissing inflation, the SB balloon is inflated at 6–10 atm and the MV stent is inflated at nominal pressure simultaneously for 10–15 s; during the second kissing inflation, a noncompliant balloon is applied to the bifurcation core and inflated simultaneously with the SB balloon to optimize stent apposition, after which the SB balloon is removed [26]. Finally, a non-balloon-based method of active SB protection is the jailed microcatheter technique (e.g., jailed Corsair), in which a microcatheter is positioned in the SB during MV stenting [27]. This creates a physical buffer at the SB ostium, maintaining access and limiting plaque shift, with the added advantage of facilitating guidewire exchange if needed.

Although the conventional passive jailed wire technique is easiest to perform, the jailed SB balloon approach improves procedural SB protection. Despite the theoretical risk of entrapment of the SB balloon by the MV stent, the incidence of entrapment was low or zero in the studies above. In the CIT-RESOLVE trial, patients with high-risk bifurcations were randomized to either the conventional jailed SB wire technique or the jailed SB balloon technique. The jailed balloon technique significantly reduced immediate SB occlusion and loss of flow [28], but a subsequent follow-up analysis found no difference in major adverse cardiac events (MACEs) after 1 year [29]. A recent meta-analysis (*n* = 1174) confirmed that active SB protection strategies, including SB jailed balloon techniques and the SB jailed Corsair technique, reduce SB deterioration during the procedure compared to the conventional passive jailed wire technique, but they do not lower rates of periprocedural myocardial infarction or improve long-term outcomes [30].

### 3.2. Three-Wire vs. Two-Wire Technique for Rewiring the SB

**Figure 3:**
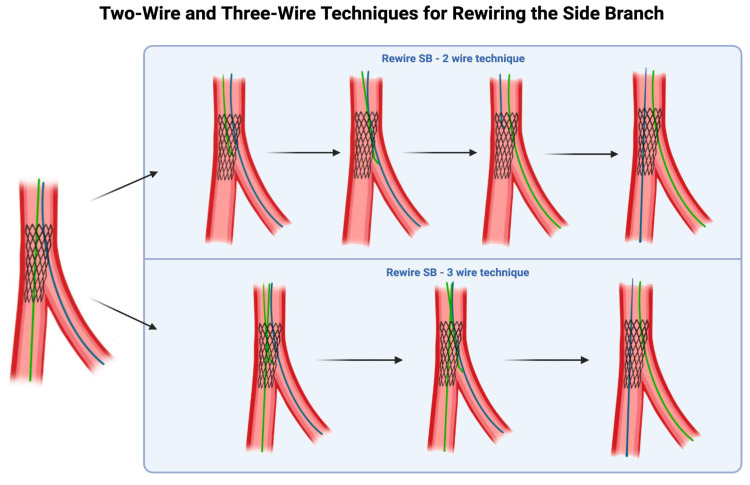
Three-wire vs. two-wire techniques for rewiring the side branch (SB). Original figure.

The standard approach for rewiring the SB involves using three wires: one in the main vessel through the lumen of the freshly deployed stent, a second in the SB jailed behind the stent, and a third wire advancing through a distal stent strut before the carina into the SB (Figure 3). However, it is possible to perform this procedure without the third wire: withdrawing the MV wire and using it to rewire the SB, leaving the jailed SB wire in place to maintain a landmark of the SB ostium.

There is variation in operator preference between these techniques. The advantage of the two-wire technique is that it ensures that the rewiring is confirmed to be within the true lumen of the proximal segment of the MV stent, unlike the introduction of a third wire, which may inadvertently engage behind a stent strut. This approach, however, requires careful and controlled withdrawal of the MV wire to ensure that it does not move proximally beyond the SB ostium, which would compromise the integrity of the technique. In contrast, the advantage of the three-wire technique is that it is more reliable, and does not risk the situation where the distal MV and the SB are both without a wire. In both cases, the jailed SB wire is only removed after wire access is regained in the SB.

The most unfavorable strategy involves withdrawing the jailed SB wire to rewire the SB; this approach fails to maintain the ostial landmark, provides no guarantee that rewiring occurs through the true lumen of the proximal stent segment, and eliminates the option for backup access should urgent inflation be required for a total SB occlusion.

### 3.3. Tips for Maintaining or Regaining SB Access

**Figure 4:**
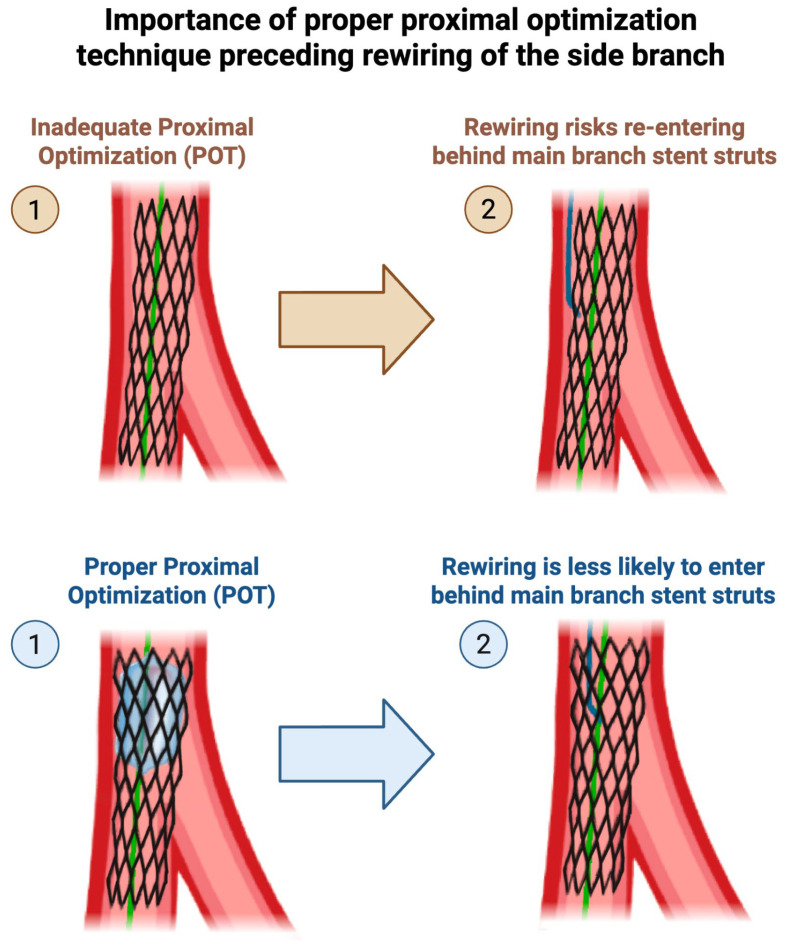
Importance of proper proximal optimization technique preceding rewiring of the side branch (SB). Original illustration.

Several strategies should be employed to increase the success of maintaining and regaining SB access. Before jailing the SB wire, partially withdraw the wire, positioning the radio-opaque segment closer to the carina, which provides a better landmark of the SB takeoff and reduces the amount of wire that needs to be pulled after jailing it (potentially reducing wire fracture). At the same time, avoid excessive withdrawal of the wire, as jailing across the radio-opaque portion of the guidewire can lead to guidewire fracture. After the MV stent is deployed, before attempting rewiring, it is important to perform adequate proximal optimization. Inadequate proximal optimization leaves a crevice in between the proximal stent struts and the proximal MV wall, into which a wire may inadvertently enter during rewiring (Figure 4).

**Figure 5:**
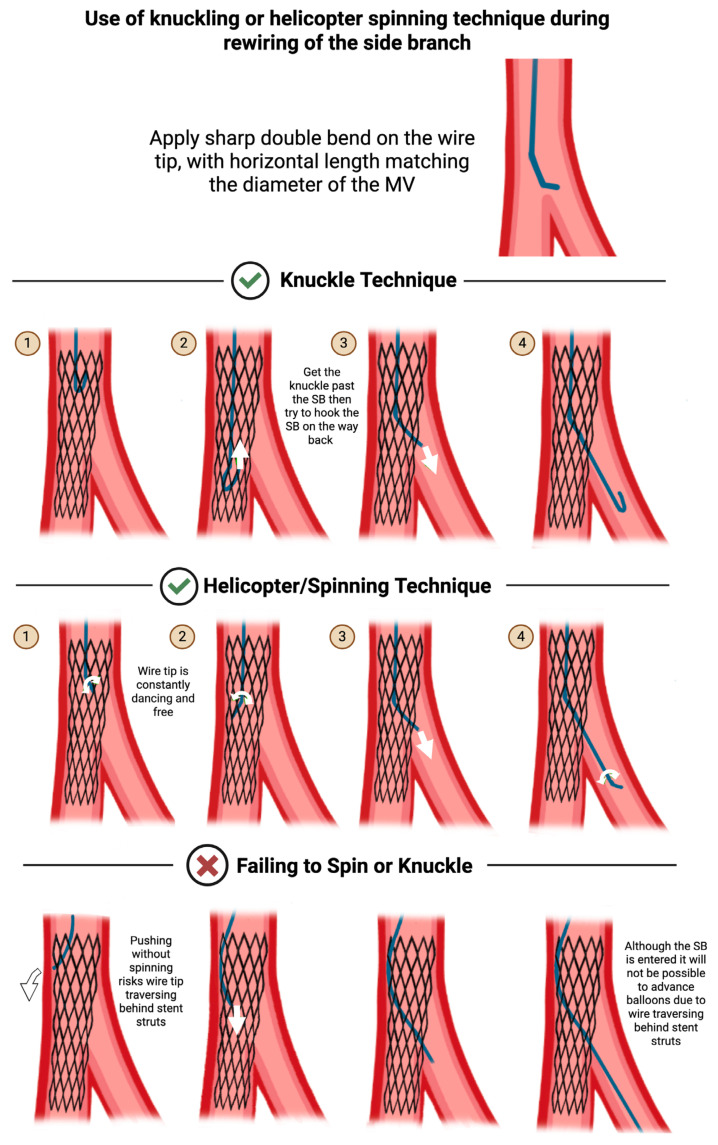
Use of the knuckling technique (**top** row) or helicopter spinning technique (**middle** row) during rewiring of the side branch (SB) will increase the likelihood of successful crossing through a distal strut. In the knuckling technique (top row), (Step 1) the tip of the wire is knuckled as it is advanced into the MV, to avoid inadvertently pushing the wire tip behind a stent strut. (Step 2) The knuckled tip is advanced past the carina into the distal MV, then (Step 3) pulled back before the carina, until the tip “drops” into the SB, after which (Step 4) it can be advanced into the SB. In the helicopter spinning technique (middle row), (Steps 1 and 2) the tip of the wire is constantly rotated clockwise and counterclockwise in alternation, to avoid inadvertently pushing the wire tip behind a stent strut. (Step 3) When the wire tip is almost at the level of carina, spinning is paused, and the wire tip is linearly directed toward the SB, after which (Step 4) it can be advanced into the SB. Failing to spin or knuckle (**bottom** row) will increase the risk that the wire tip may traverse behind a stent strut, which increases the risk of subsequent inability to cross with a balloon. Original illustration.

There are two acceptable approaches for increasing the likelihood of successful crossing through a distal strut: the knuckling technique and the helicopter spinning technique (Figure 5).

In the knuckling technique, allow the tip of the wire to form a knuckle as you advance into the MV. The knuckled tip avoids inadvertently pushing the wire tip behind a stent strut. Advance the knuckled wire tip past the carina into the distal MV, then pull back before the carina until the tip “drops” into the SB, then advance through the stent strut.

In the helicopter spinning technique, constantly apply a rotational force on the wire (alternating clockwise and counterclockwise) such that the tip of the wire is perpetually “dancing”. The dancing tip avoids inadvertently pushing the wire tip behind a stent strut. When the wire tip is almost at the level of the carina, switch from spinning to linearly directing the tip toward the SB, then advance the wire through the stent strut.

In both cases, the likelihood of successful rewiring can be facilitated by applying a sharp double bend on the wire tip, with the horizontal length matching the diameter of the MV. To confirm that the wire has recrossed through a distal stent strut, optical coherence tomography (OCT) can be employed for visualization.

**Figure 6:**
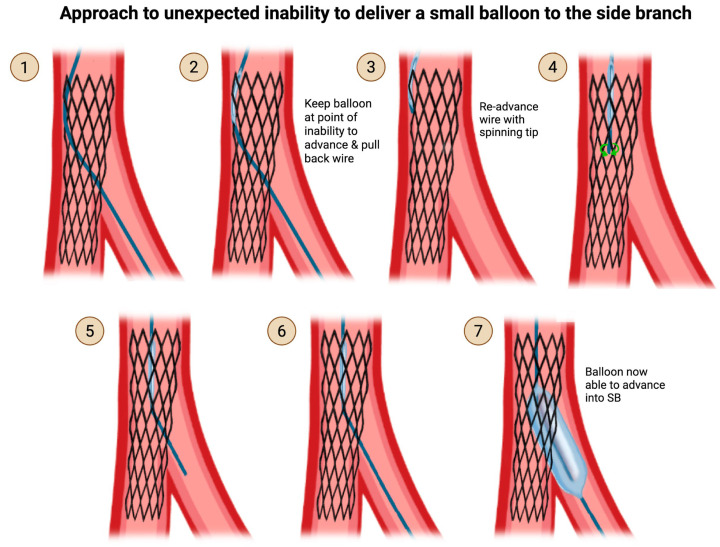
Approach to unexpected inability to deliver a small balloon to the side branch (SB). If a balloon (Step 2) cannot be crossed into the SB, even after downsizing to a 1.0 mm balloon, that suggests (Step 1) the guidewire may have inadvertently passed behind the struts of a stent. In this situation, (Step 3) keep the balloon positioned at the point of inability to advance, and withdraw the wire to that point, effectively wihdrawing the wire from behind the stent struts. (Step 4) Re-advance the wire with a spinning tip, and (Steps 5 and 6) re-cross the SB, with subsequent (Step 7) ability to deliver and inflate the balloon in the SB. Original illustration.

If a balloon cannot be crossed into the SB, even after downsizing to a 1.0 mm balloon, it may be possible that the guidewire has passed inadvertently behind the struts of a stent. In this situation, keep the balloon positioned at the point of the inability to advance, and withdraw the wire to that point. This should effectively withdraw the wire from behind the stent struts. Re-advance the wire with a spinning tip, and recross the SB (Figure 6). If you are certain that the guidewire is in the true lumen and not entrapped, you may advance a microcatheter through the recrossed SB to facilitate the subsequent delivery of a balloon for high-pressure post-dilation to open the stent struts into the SB.

### 3.4. Guidewire Entrapment in the SB

In the event of guidewire entrapment, it is essential to avoid applying force to extract the guidewire, as this may lead to fracture. Several approaches may be employed to resolve the issue: (a) Apply continuous low traction. (b) Utilize a torquer to rotate the trapped guidewire out of its entrapment. (c) Introduce a small balloon or microcatheter to the site of entrapment and gently withdraw. (d) Advance a small balloon behind the stent struts through the jailed guidewire and inflate to release the trapped wire. If employing this technique, it is crucial to re-perform post-dilatation (POT) to ensure that the proximal segment of the stent remains fully expanded.

## 4. Algorithm for Bifurcation PCI

**Figure 7:**
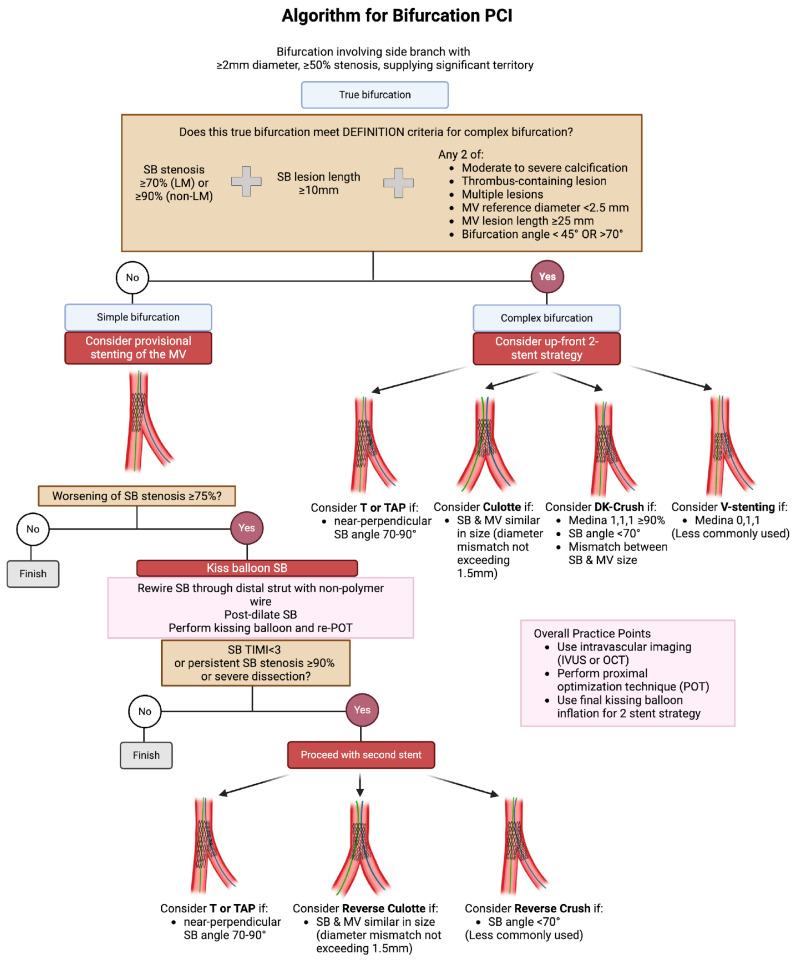
Algorithm for bifurcation percutaneous coronary intervention (PCI).

Synthesizing the concepts described in this paper as well as its companion paper [2], decision-making in bifurcation PCI can be simplified as follows (Figure 7). The first decision point is whether the lesion is simple or complex. Using the DEFINITION criteria, complex bifurcations are defined by the presence of severe SB stenosis (≥70% for left main, ≥90% for non-left main) combined with extensive SB disease (≥10 mm length), plus any two additional high-risk features: moderate-to-severe calcification, thrombus burden, multiple lesions, small MV reference diameter (<2.5 mm), long MV lesions (≥25 mm), or challenging bifurcation angles (<45° or >70°).

For non-complex bifurcations, a provisional strategy is recommended. The rationale for MV stenting with conditional SB intervention is rooted in multiple randomized trials showing similar outcomes to systematic two-stent strategies but with shorter procedures, less contrast, and lower periprocedural risk. If the SB stenosis worsens beyond ≥75%, rewire through a distal strut using a non-polymer wire, perform post-dilation through the stent struts into the SB, and perform kissing balloon inflation, with optional proximal re-optimization technique (re-POT). If the SB becomes severely compromised (TIMI < 3 flow, ≥90% stenosis, or severe dissection), then proceed with a second stent. This approach maximizes the benefits of single-stent simplicity while providing clear criteria for escalation when anatomical or functional results are suboptimal.

For complex bifurcations, an upfront two-stent strategy is recommended. Here, the choice of technique is tailored to lesion morphology: T or TAP stenting for near-perpendicular SB angles (70–90°), Culotte for vessels with similar reference diameters (diameter mismatch < 1.5 mm), DK crush for complex left main bifurcations with Medina 1,1,1 classification, acute angles (<70°) or significant vessel size mismatch, and V-stenting for Medina 0,1,1 lesions, though this latter technique is less commonly employed.

Throughout both pathways, several overarching practice points should be kept in mind. Intravascular imaging (IVUS or OCT) should be used liberally, particularly in left main or complex bifurcations, to guide stent sizing, confirm adequate expansion, and assess SB ostial disease. Proximal optimization ensures that the main vessel stent conforms to its natural taper and facilitates easy, safe access to the SB. Final kissing balloon inflation, while not always mandatory in simple provisional cases, is essential in all two-stent strategies to minimize strut distortion and optimize carina geometry.

## 5. Outcomes of Randomized Trials

### 5.1. Synopsis of Landmark Trials of Provisional vs. Two-Stent Strategies

Provisional stenting is commonly used as the first-line strategy for most bifurcation lesions based on simplicity, shorter procedure time, and generally non-inferior outcomes. In the mid-2000s, numerous trials focused on comparing one- vs. two-stent strategies for coronary bifurcation lesions (Table 2). At the time, many believed that treating both the MV and the SB with stents would yield better outcomes. The NORDIC I trial (2006) was among the first of the landmark randomized controlled trials (RCTs) [31]. It randomized 413 patients with non-left main bifurcation lesions to planned two-stent techniques versus provisional stenting [31]. At 6 months, there was no statistically significant difference in MACEs (3.4% vs. 2.9%) between two groups [31]. However, the two-stent group had longer procedure time, longer fluoroscopy time, and more contrast use [31].

In 2008, the BBK (Bifurcations Bad Krozingen) trial also supported this approach [32]. BBK compared routine T-stenting of the SB versus provisional T-stenting in 202 patients [32]. The incidence of side-branch restenosis at 9 months (27.7% vs. 23%, *p* = 0.15) and one-year target lesion revascularization (8.9% vs. 10.9% *p* = 0.64) were statistically similar, with a trend toward more main-vessel restenosis in the two-stent group [32]. BBK was angiography-driven and not powered for clinical events.

In 2009, the CACTUS (Coronary bifurcations: Application of the Crushing Technique Using Sirolimus-eluting stents) trial investigated the crush two-stent technique versus provisional stenting in true bifurcations in 350 patients in 12 European centers [33]. Of note, CACTUS mandated final KBI in both arms. It found no significant difference in MACE (15.0% vs. 15.8%, *p* = 0.95) and restenosis rates (MV restenosis 4.6% vs. 6.7% and SB restenosis 13.2% vs. 14.7%, *p* = NS) at 6-month follow-up [33].

A year later, in 2010, the BBC ONE trial (British Bifurcation Coronary study: Old, New, and Evolving strategies) also reinforced this concept. BBC ONE randomized 500 patients (82% with true bifurcations) to either a provisional stenting strategy (with kissing balloon inflation optional) or a planned two-stent strategy (crush or Culotte with mandatory final kissing balloon) [34]. At 9-month follow-up, there was significantly lower MACEs associated with provisional stenting compared to planned two-stent approaches (8% vs. 15.2%, *p* = 0.009) [34]. Notably, the two-stent group had a higher incidence of myocardial infarction (11.2% vs. 3.6%, *p* = 0.001) [34]. The results of the BBC ONE trial solidified provisional stenting as the standard of care for non-left main bifurcations [34].

These trials influenced cardiology guidelines to endorse a provisional stenting approach, with subsequent implantation of a second stent in the SB only if there is significant residual stenosis or flow compromise after MV stenting. The 5-year follow-up combined data of the NORDIC I and BBC ONE trials became available in the mid-2010s and showed that 5-year mortality was significantly lower in the provisional stenting group compared to the systematic two-stent group (3.8% vs. 7.0%, *p* = 0.04) [38]. It should be noted that most early trials used first-generation drug-eluting stents (DESs), which had thicker struts and were less flexible, potentially exacerbating side-branch compromise or stent thrombosis. Newer generation stents have thinner struts, better deliverability, and side-branch access. A meta-analysis of one- vs. two-stent strategies in the context of newer-generation DESs found no difference in MACEs but a persistent higher periprocedural MI risk with planned two-stents [39].

Several major trials have shown that a provisional strategy is associated with similar outcomes to an upfront two-stent strategy even in true bifurcations, particularly the non-complex non-LM true bifurcations: PERFECT, EBC TWO, and NORDIC IV. In 2015, the PERFECT (Optimal Stenting Strategy for True Bifurcation Lesions) trial randomized 419 patients to provisional stenting versus two-stent crush. After 1 year, the MACE rate did not differ (18.5 vs. 17.9%, *p* = 0.84) [35]. In 2016, the EBC TWO trial randomized 200 patients with non-LM true bifurcation lesions to provisional T-stenting versus planned two-stent Culotte. At 5 years, the primary composite endpoint (which was not reported as the MACE rate) of death, myocardial infarction, and target vessel revascularization did not differ (7.7% vs. 10.3%, *p* = 0.53), while procedure time, X-ray dose, and cost favored provisional T-stenting [36]. In 2020, the NORDIC IV trial randomized 450 patients with non-LM true bifurcation lesions to provisional versus two-stent techniques (Culotte was recommended, but the specific technique was left to the operator’s discretion). At two years, the rate of MACEs did not differ (12.9% vs. 8.4%, *p* = 0.12), while procedure time, X-ray dose, and contrast dose favored provisional stenting [37]. In conclusion, EBC TWO, PERFECT, and NORDIC IV showed that even for true bifurcations, there was no significant difference in MACEs between provisional and two-stent strategies, and provisional stenting was associated with less procedure time, fluoroscopic dose, and contrast use.

### 5.2. Synopsis of Landmark Trials Focusing on Complex Bifurcations

As lesion complexity being treated by PCI increased, investigators began to differentiate which two-stent strategies could add value (Table 3). Even though a provisional single-stent strategy was recommended as a default strategy, it was felt that the more complex lesions might require an upfront two-stent approach, especially a large, diseased SB or a very tight, long lesion in the SB (“true” Medina 1,1,1 lesions with heavy disease burden), which often required two stents eventually. The DKCRUSH-I Bifurcation Study in 2008 demonstrated that the DK crush technique has the potential of increasing side-branch stent opening, minimizing residual crush at the carina, and reducing the rate of stent thrombosis and TLR as compared to the classical crush [40].

While DKCRUSH-I (2008) compared DK crush with classical crush, the DKCRUSH-II trial (2011) compared DK crush with provisional stenting in 370 patients with a majority of non-left main true bifurcation lesions [41]. At 1-year follow-up, there was no significant difference in the MACE rate (10.3% DK vs. 17.3% provisional, *p* = 0.07) [41]. However, the DK crush technique had lower TLR (4.3% DK vs. 13.0% provisional, *p* = 0.005) and lower side-branch restenosis (4.9% DK vs. 22.2% provisional, *p* < 0.001) at 8-month follow-up [41]. After 5 years, the DK crush technique continued to show lower rates of target lesion revascularization compared to provisional stenting, although the MACE rate remained comparable [42]. This was the beginning of a new era and shaped clinical practice; not all two-stent strategies were equal—a carefully executed DK crush for complex lesions (large SB with extensive disease) could achieve excellent patency in both branches. Hence, DK crush emerged as the favored two-stent technique for complex bifurcations with large SBs and long ostial SB disease [42].

### 5.3. Synopsis of Landmark Trials Focusing on Left Main Bifurcations

LM bifurcation lesions were a focus of subsequent trials (Table 4). Early trials such as Nordic I or BBC ONE either excluded LM cases or had only a small subset of LM disease. The DKCRUSH-III trial (2015) was one of the first to study left main bifurcations comparing the DK crush technique and the Culotte technique (both arms using two stents) in distal unprotected left main lesions [43]. After 3 years, DK crush was superior to Culotte, with lower MACE, target vessel revascularization, and stent thrombosis rates [43]. However, it has been noted that outcomes in the Culotte arm were numerically worse than outcomes in European cohorts such as EBC TWO or EBC MAIN, which predominantly used Culotte in the two-stent arm, raising the question of whether proficiency in the DK crush technique or Culotte technique may confound outcomes.

The DKCRUSH-V trial (2017) investigated a similar question to DKCRUSH-II but focused on left main bifurcation disease specifically. It randomized 482 patients with true distal left main bifurcation disease (Medina 1,1,1 or 0,1,1) to provisional stenting versus a planned DK crush two-stent strategy [14]. At 1-year follow-up, DK crush was associated with a significant reduction in the primary outcome of target lesion failure, defined as the composite of cardiac death, target-vessel MI, or target lesion revascularization (5% DK vs. 10.7% provisional, *p* = 0.02) [14]. The approximately 50% relative risk reduction in adverse events in the DK crush arm was mainly driven by significant decreases in target-vessel MI (2.9% provisional vs. 0.4% DK, *p* = 0.03) and stent thrombosis (3.3% provisional vs. 0.4% DK, *p* = 0.02) [14].

The EBC MAIN trial (European Bifurcation Club Left Main Study) of 2021 was a trial that randomized 467 patients to either a provisional approach or a two-stent strategy [44]. The method of two-stent technique was at the discretion of the operator. Unlike in DKCRUSH-III, Culotte was most commonly used in EBC MAIN, and very few cases involved DK crush [44]. At 3-year follow-up, EBC MAIN found no statistically significant difference in the MACE rate between the two groups (23.5% provisional vs. 29.5% two-stents, *p* = 0.11) [45]. Rates of all-cause mortality (10% provisional vs. 13% two-stents) and MI (12% provisional vs. 11% two-stents) were similar between strategies [45]. However, target lesion revascularization (TLR) was significantly lower in the provisional arm (8.3% provisional vs. 15.6% two-stents, *p* = 0.013) [45]. Subgroup analysis revealed that the provisional technique was favorable when the side-branch was smaller than 3.25 mm or had <10 mm lesion length, whereas very large, long side-branch lesions fared better with two stents [45]. EBC MAIN concluded that provisional remains the default approach for simple LM disease. While DKCRUSH-V yielded excellent outcomes, EBC MAIN may have been more representative of real-world practice, with a mix of techniques and operators over a longer term.

Because of ongoing debate on when an upfront two-stent strategy is truly indicated, the DEFINITION II trial (2020) was conducted [13]. In DEFINITION, complex bifurcation lesions were defined to include factors such as Medina 1,1,1/0,1,1/1,0,1, SB diameter > 2.5 mm, lesion length > 10 mm, and severe calcification (see Table 1) [13]. DEFINITION II randomized 653 patients to a systematic two-stent strategy (77.8% DK crush in that arm) versus provisional stenting [13]. At 1-year follow-up, the two-stent strategy was associated with a significantly lower target lesion failure (the composite of cardiac death, MI, or target revascularization) than the provisional group in high-risk bifurcations [13]. The DEFINITION II provided evidence that in truly complex bifurcation lesions, an upfront two-stent approach is beneficial. For that reason, the bifurcation complexity assessment is strongly recommended.

In conclusion, provisional stenting remains the first strategy for non-left main and simple bifurcations, as evidenced by the collective outcomes of these landmark trials, while an upfront two-stent strategy is appropriate for left main or complex bifurcations meeting the DEFINITION criteria. Numerous ongoing trials will provide further clarity into subsets of bifurcation anatomies that benefit from specific techniques (Supplementary Table S1). While there are competing results regarding the superiority of individual two-stent techniques such as Culotte or DK crush, the more important point is that proper lesion preparation, proper POT, proper kissing balloon inflation, and proper guidance with IVUS/OCT are paramount to the success of the two-stent strategy, more so than the exact technique used. The adoption in technologies such as intravascular imaging may be very helpful and play an important role in decision-making.

## 6. Evolving Techniques

### 6.1. Role of Intravascular Imaging in Bifurcation PCI

In general PCI, intravascular ultrasound (IVUS) is used for the assessment of lesion characteristics, optimization of stent deployment, optimal stent expansion, exclusion of edge plaque burden >50%, and edge dissection [46]. In bifurcation PCI, IVUS is useful in both the MV and SB, as it facilitates adequate lesion preparation of SB and MV; accurate measurement of distal MV diameter for 1:1 sizing the MV stent, subsequent stent post-dilation, and subsequent kissing balloon inflation; accurate measurement of proximal MV diameter for 1:1 sizing subsequent POT and rePOT; and accurate measurement of SB diameter for 1:1 sizing for stenting and kissing balloon inflation if necessary [47].

OCT can provide information for the aforementioned assessments, plus a key additional assessment: 3D-OCT is uniquely helpful for confirming distal-cell recrossing, associated with better strut configuration and larger SB ostial area after kissing [48]. In provisional stenting, wire crossing through distal cells is preferred when performing KBI or T/TAP stenting. In the DK crush technique, wire crossing through non-distal (proximal or central) cells is recommended for both the first KBI and the second KBI [14,49]. OCT’s strut-level view helps avoid abluminal recrossing and can visualize adequacy of crush [50]. This assessment is more challenging in IVUS.

What is the randomized trial data for intravascular imaging (IVI) in bifurcation PCI? A 3-year follow-up of the ULTIMATE (Intravascular Ultrasound Guided Drug-eluting Stents Implantation in All-Comers Coronary Lesions, including patients with CBL) trial showed a significant reduction in target vessel failure (TVF) with IVUS in the bifurcation subgroup (hazard ratio [HR] 0.48, 95% CI: 0.27–0.87), in accordance with the finding in the overall trial population [51]. The 5-year results from DKCRUSH-II identified a reduced rate of MI in patients who underwent IVUS assessment (1.8% vs. 5.4%; *p* = 0.043) [42]. In the IVUS sub-study of NOBLE, 5-year the MACE rate was not significantly different with respect to whether IVUS was performed or not (18.9% vs. 25.0%, *p* = 0.45), but it should be noted that IVUS was not performed in a randomized manner [52]. In one major study of IVUS-guided LM Bifurcation PCI using the crush technique, the minimal stent area thresholds that best predicted freedom from 5-year MACEs was 11.8 mm^2^ for the distal LM, 8.3 mm^2^ for the ostial LAD, and 5.7 mm^2^ for the ostial LCX [53].

The OCTOBER (European Trial on Optical Coherence Tomography Optimized Bifurcation Event Reduction) trial randomized 1,201 patients with bifurcation disease to OCT-guided PCI versus angiography-guided PCI [48]. Patients in the OCT-guided PCI arm had lower MACEs at 2 years (10.1% vs. 14.1%, CI: 0.50–0.98%). A recent meta-analysis of seven trials, incorporating the bifurcation PCI subgroups of larger IVI trials, found that IVI guidance was associated with a significant reduction in target vessel failure in bifurcation lesions (RR 0.70, CI: 0.53 to 0.92) [47].

The ongoing DKCRUSH-VIII trial will shed light on the impact of IVUS-guided vs. angiography-guided bifurcation PCI using the DK crush technique [54]. The EBC concludes that while IVUS and OCT are increasingly taking on a central role in bifurcation PCI, angiography continues to remain the principal modality for guiding bifurcation PCI at this time [50].

### 6.2. Role of Drug-Coated Balloons in SB Treatment

Drug-coated balloons (DCBs) offer homogenous administration of high concentrations of antiproliferative drugs without a permanent scaffold or polymer, and have found a central role in the treatment of in-stent restenosis. The “leave nothing behind” philosophy advocates for PCI without leaving any permanent foreign object, as it avoids the chronic inflammation, neoatherosclerosis, or stent thrombosis that can occur with metal stents [55]. In bifurcation disease, DCBs offer specific advantages. In bifurcation lesions that call for provisional stenting, DCBs can treat small caliber SBs without the need to add a SB stent. DCB also allows bifurcation PCI to be accomplished without the risk of incomplete SB ostial coverage or stent deformation, malapposition, or fracture, which may lead to thrombosis and restenosis.

The main application of DCB that has been explored in bifurcation PCI is its use for small SBs. In BEYOND (2022), which randomized 222 patients with true bifurcation to DCB vs. POBA using a compliant balloon for the SB after provisional stenting of the MV, the % stenosis of the SB was marginally lower (29% vs. 40%), and there was no difference in clinical events such as MACEs at 9 months [56]. In DCB-BIF (2024), which randomized 784 patients with true bifurcation to DCB vs. POBA using a non-compliant balloon for the SB after provisional stenting of the MV, the 1-year MACE rate was significantly reduced (7.2% vs. 12.5%, *p* = 0.013) in the DCB arm, driven by lower target-vessel MI from DCB [57]. DCB-BIF is the first trial to show a clinical event reduction from DCB in the SB. One could use the results from DCB-BIF to support the use of DCB for treating SB pinching after MV stenting. But it is important to keep in mind that in DCB-BIF, rates of TLR were low (<1.5%) and did not differ significantly between the DCB and POBA arms. Instead, the observed reduction in MI in the DCB arm was driven by fewer periprocedural MIs and early spontaneous MIs, occurring within days of the procedure. After the first few days, the event curves run almost parallel, raising doubt about whether this reflects a true mechanistic effect of DCB or represents a chance finding. Unfortunately there are no robust trials directly comparing DCB of the SB with stenting of the SB.

While promising, optimal lesion preparation, appropriate sizing, patient selection, and head-to-head comparisons against modern DES in bifurcations are still required to define the precise role of DCBs in contemporary bifurcation PCI strategies, given that the preponderance of DCB trials in bifurcation PCI have used POBA as their comparator arm. At this time, there is no official recommendation from the EBC or other major guideline societies about specific bifurcation scenarios where DCBs are clearly indicated.

### 6.3. Role of Bifurcation-Dedicated Stents

Bifurcation-dedicated stents (BDSs) have been developed to address the persistent technical challenges of conventional bifurcation techniques, including (i) rewiring the SB after provisional stenting, (ii) delivering equipment across the jailing stent struts, (iii) distorting the MB stent by SB dilation, and (iv) fully covering the SB ostium [58].

The design of dedicated bifurcation stents can be divided into four categories. The first category involves main vessel stents with SB access, including the Nile PAX stent (Minvasys, Genevilliers, France), Twin Rail (Invatect S.r.l., Brescia, Italy), Petal (Boston Scientific, Natick, MA, USA), Multilink Frontier (Abbott Vascular Devices, Santa Clara, CA, USA/Guidant Corporation, Santa Clara, CA, USA), Trieme (Trireme Medical Inc., Pleasanton, CA, USA), Side-kick (Y-med Inc., San Diego, CA, USA), BiOSS (Balton, Warsaw, Poland), and Stentys (Stentys S.A.S., Clichy, France) (Supplementary Table S2). The second category involves SB stents with main vessel access, including the Capella Side Guard (Capella Inc., Cambridge, MA, USA) and Tryton (Tryton Medical, Newton, MA, USA). The third category involves proximal main vessel stents, including the Axxess (Devax, Irvine, CA, USA). The fourth category involves bifurcated Y-stents, including the Medtronic Bifurcated Stent (Medtronic, Santa Rosa, CA, USA) and Advanced Bifurcation System (ABS, Los Angeles, CA, USA) (Supplementary Table S3).

One common feature of these BDSs is delivery over two wires. The advantage is maintaining dual-wire access in the MB and the SB. Many of these devices minimize or eliminate the need for final KBI, either by design, as in the self-expanding Axxess stent, or by preserving the geometric integrity during post-dilation, as in the STENTYS stent [59].

Nevertheless, there are a few disadvantages to BDS. The SB wire often curves away to the wall opposing the SB, making SB alignment challenging. This is known as guidewire bias. Secondly, wire wrap can occur when the two wires entangle distal to the stent delivery system. Third, the dual lumen required for the two-wire delivery system is often bulky, requiring guide catheters larger than 6Fr and making delivery and torque transmission difficult, particularly in calcified and tortuous vessels. With the approval of DCBs in the United States and the increasing adoption of DCBs globally, the role of BDSs may diminish in the coming decades.

### 6.4. Role of Bioresorbable Vascular Scaffolds and Bioadaptor Stents

Bioresorbable vascular scaffolds (BRSs) are bioprostheses that provide transient support to the vessel wall without leaving behind a permanent metallic cage, hence the use of the term “scaffold” instead of “stent” [60]. BRSs are made of self-degrading materials such as poly-L-lactide (PLLA), which gradually dissolve over 2–3 years, restoring native vessel vasomotion [61]. They were designed to overcome the limitations of traditional metallic cage stents, which impair arterial physiology, limit future surgical and transcatheter options, and are susceptible to very late stent thrombosis [62]. The most well-studied of these is the Absorb BVS from Abbott (Santa Clara, CA, USA), which received CE mark in 2012 [63].

Unfortunately, these theoretical advantages have not translated into reproducible clinical advantages. BRSs suffer from poor deliverability due to scaffold thickness and suboptimal acute gain. Residual under-expansion and malapposition is harder to fix in BRSs compared to DESs. The thick-strut polymeric scaffolds of BRSs make recrossing of the side branch harder. Aggressive side-branch or post-carinal inflations can easily risk scaffold deformation and disruption. Consequently, BRSs have higher target lesion failure and device thrombosis rates compared to DESs, as demonstrated in a meta-analysis of individual patient-level data at 5 years [64].

In response to these limitations, bioadaptor stents were developed. The DynamX bioadaptor stent (Elixir Medical, Milpitas, CA, USA) functions as a conventional DES over the first 6 months, providing structural support and releasing sirolimus to prevent restenosis. After six months, the polymer base coat dissolves, and the helical strands unlock from each other, allowing the metal strands to shift and adapt to the vessel’s natural motion, restoring vessel pulsatility and compliance. One-year results from randomized trials show that bioadaptor stents are non-inferior to conventional DESs in target lesion failure, with improved compliance and cyclic pulsatility, and lower late lumen loss [65,66]. It remains to be seen whether bioadaptor stents can demonstrate benefit in bifurcation PCI specifically, and whether the 1-year results translate into similar long-term outcomes.

## 7. Conclusions

The management of bifurcation lesions has evolved significantly. Intravascular imaging and physiologic testing serve as valuable adjuncts, enhancing lesion characterization, optimizing procedural planning, and guiding real-time decision-making. In this second part of our two-part review, we have highlighted advanced considerations that refine procedural planning and strategy selection—ranging from predictors of SB compromise to landmark trial data evaluating technique outcomes. While provisional stenting remains the default for many, select lesions clearly benefit from upfront two-stent strategies, particularly when informed by imaging data. Future directions will likely involve increased integration of physiology-based decision tools, imaging-guided optimization, and novel device technologies, including increasing adoption of DCB-based SB strategies.

## Figures and Tables

**Table 1:** DEFINITION criteria classification for complex bifurcation. A bifurcation is considered complex if it meets one major criterion + any two minor criteria.

Major Criteria	Minor Criteria
For LM bifurcations: SB lesion length of ≥10 mm, with diameter stenosis of SB ≥ 70% For non-LM bifurcations: SB lesion length of ≥10 mm, with diameter stenosis of SB ≥ 90%	Moderate to severe calcification Thrombus-containing lesion Multiple lesions MV reference diameter < 2.5 mm MV lesion length ≥ 25 mm Bifurcation angle < 45° or >70°

**Table 2:** Select landmark trials in bifurcation percutaneous coronary intervention (PCI) evaluating the risk of major adverse cardiovascular events (MACEs).

Trial	Strategy Compared	Follow-Up	MACE	Definite/Probable IST	ISR/TLR
Nordic Bifurcation (2006) [31]	Provisional vs. two-stent (Culotte or Crush)	6 mo	2.9% vs. 3.4%, *p* = NS	0.5% vs. 0%, *p* = 0.31	ISR 5.3% vs. 5.1%, *p* = NS TLR 1.9% vs. 1.0%, *p* = 0.36
BBK (2008) [32]	Provisional vs. routine T-stenting	12 mo	12.9% vs. 11.9%, *p* = 0.83	2% vs. 2%, *p* = 1.0	TLR 10.9% vs. 8.9%, *p* = 0.64
CACTUS (2009) [33]	Provisional vs. two-stent (Crush)	6 mo	15% vs. 15.8%, *p* = 0.95	1.1% vs. 1.7%	TLR 6.3% vs. 7.3%, *p* = 0.83
BBC ONE (2010) [34]	Provisional vs. two-stent (Culotte or Crush)	9 mo	8% vs. 15.2%, *p* = 0.009	0.4% vs. 2.0%	TLR 5.6% vs. 6.8%, *p* = NS
PERFECT 2015 [35]	Provisional vs. two-stent (Crush)	12 mo	18.5% vs. 17.8%, *p* = 0.85	0% vs. 0.5%, *p* = 0.32	ISR 11% vs. 8.4%, *p* = 0.44 TLR 3.4% vs. 1.9%, *p* = 0.33
EBC TWO 2016 [36]	Provisional vs. two-stent (Culotte) in non-LM	12 mo	7.7% vs. 10.3%, *p* = 0.53	0.97% vs. 2.06%, *p* = 0.357	TVR 2.9% vs. 1.0%, *p* = 0.621
NORDIC IV 2020 [37]	Provisional vs. two-stent (Culotte)	24 mo	12.9% vs. 8.4%, *p* = 0.12	2.8% vs. 2.2%, *p* = 0.7	TLR 9.2% vs. 6.2%, *p* = 0.23

In-stent thrombosis (IST), in-stent restenosis (ISR), target lesion revascularization (TLR), and target vessel revascularization (TVR) after provisional versus two-stent bifurcation stenting strategies.

**Table 3:** Select landmark trials in bifurcation percutaneous coronary intervention (PCI) evaluating the risk of major adverse cardiovascular events (MACEs).

Trial	Strategy Compared	Follow-Up	MACE	Definite/Probable IST	ISR/TLR
DKCRUSH-I (2008) [40]	Classical vs. DK crush	8 mo	24.4% vs. 11.4%, *p* = 0.02	3.2% vs. 1.3%, *p* = 1.0	22.6% vs. 9%, *p* = 0.03
DKCRUSH-II (2011) [41]	Provisional vs. two-stent (DK crush) in non-LM	12 mo	17.3% vs. 10.3%, *p* = 0.07	1.1% vs. 2.7%, *p* = 0.449	TLR 13% vs. 4.3%, *p* = 0.005

In-stent thrombosis (IST), in-stent restenosis (ISR), target lesion revascularization (TLR), and target vessel revascularization (TVR) after crush-based stenting in complex bifurcations.

**Table 4:** Select landmark trials in bifurcation percutaneous coronary intervention (PCI) evaluating the risk of major adverse cardiovascular events (MACEs).

Trial	Strategy Compared	Follow-Up	MACE	Definite/Probable IST	ISR/TLR
DKCRUSH-III (2015) [43]	DK crush vs. Culotte (LM bifurcation) in unprotected LM	36 mo	8.2% vs. 23.7%, *p* < 0.001)	0.5% vs. 3.9%, *p* = 0.02	TLR 5.8% vs. 18.8%, *p* < 0.001
DKCRUSH-V (2017) [14]	Provisional vs. two-stent (DK crush-LM bifurcation) in distal LM	12 mo	10.7% vs. 5%, *p* = 0.02	3.3% vs. 0.4%, *p* = 0.02	TLR 7.9% vs. 3.8%, *p* = 0.06
DEFINITION II (2020) [13]	Provisional vs. two-stent (DK crush) in complex bifurcations	12 mo	11.4% vs. 6.1%, *p* = 0.019	2.5% vs. 1.2%, *p* = 0.244	TLR 5.5% vs. 2.4%, *p* = 0.049
EBC MAIN (2021) [44]	Provisional vs. two-stent (Culotte or TAP) in LM	12 mo	14.7% vs. 17.7%, *p* = 0.34	1.7% vs. 1.3%, *p* = 0.9	TLR 6.1% vs. 9.3%, *p* = 0.16

In-stent thrombosis (IST), in-stent restenosis (ISR), target lesion revascularization (TLR), and target vessel revascularization (TVR) in left main bifurcations.

## Data Availability

No new data were created or analyzed in this study. Data sharing is not applicable to this article.

## Supplementary Materials

The following supporting information can be downloaded at https://www.mdpi.com/article/10.3390/jcdd12110439/s1, Table S1: Ongoing bifurcation stenting trials on clinicaltrials.gov. Table S2: Bifurcation-dedicated stents built as a main vessel stent with side branch access. Table S3: Bifurcation-dedicated stents built as a side branch stent with main vessel access; proximal main vessel stent; or pre-made bifurcation stent. References [67,68,69,70,71,72,73,74,75,76,77,78,79,80,81,82] are cited in the Supplementary Materials.
