# Supplementary material for: Coronary Bifurcation PCI—Part II: Advanced Considerations
Source: J Cardiovasc Dev Dis. 2025 Nov 6;12(11):439. doi: 10.3390/jcdd12110439 (PMC12653562; doi:10.3390/jcdd12110439)
Supplement: Supplementary file 1 [file jcdd-12-00439-s001.zip › jcdd-3927754-supplementary.pdf]

Supplement to: Advanced Considerations in Coronary Bifurcation PCI

Rongras Damrongwatanasuk[1], Sara Pollanen[2], Ju Young Bae[3], Jason Wen[4], Michael G. Nanna[5], Abdulla Al-Damluji[6], Mamas Mamas[7], Elias B Hanna[8], Jiun-Ruey Hu[4]

1. Department of Internal Medicine, Division of Cardiovascular Medicine, University of Louisville, Kentucky, United States https://orcid.org/0009-0004-9829-9764
2. University of Toronto, Temerty Medicine, Toronto, Ontario, Canada. https://orcid.org/0000-0003-4953-2414
3. Department of Internal Medicine, Division of Cardiovascular Medicine, Weill Cornell Medicine, New York Presbyterian Hospital, New York, NY, United States. https://orcid.org/0000-0001-8509-7099
4. Department of Cardiology, Smidt Heart Institute, Cedars-Sinai Medical Center, Los Angeles, CA, United States. ORCID: https://orcid.org/0009-0000-1130-1691; https://orcid.org/0000-0003-1390-508X
5. Department of Medicine, Section of Cardiovascular Medicine, Yale New Haven Hospital, New Haven, CT, United States
6. Cardiovascular Center on Aging, Department of Cardiovascular Medicine, Cleveland Clinic Foundation, Cleveland, OH, United States. https://orcid.org/0000-0002-8774-6416
7. Keele Cardiovascular Research Group, Keele University, Newcastle ST5 5BG, United Kingdom
8. Cardiology Department, Lebanese American University, Beirut, Lebanon. https://orcid.org/0000-0002-0967-9115

Supplementary Table S1: Ongoing bifurcation stenting trials on clinicaltrials.gov

Study Title (NCT Number)
Bifurcation Stenting Using 2 Link Stent Nobori Versus 3 Link Stent Xience (NCT01574586)
Culotte Versus DK-CRUSH Technique in Non-left Main Coronary Bifurcation Lesions (NCT04192760)
COmplex Bifurcation PCI: AXXESS Device + Absorb BVS, vs Modified T Stenting With Absorb BVS (NCT02628288)
IVUS-guided DK Crush Stenting Technique for Patients With Complex Bifurcation Lesions (NCT03770650)
Modified T-Stenting With Szabo Technique Versus T-Stenting for Bifurcation Lesions in Coronary Heart Diseases (NCT03714802)
The European Bifurcation Club Left Main Study (NCT02497014)
The Nordic-Baltic Bifurcation Study IV (NCT01496638)
Tryton Post Approval Study (PAS) for the Tryton Side Branch Stent (NCT03318796)
Keep bIfurcation Single Stenting Simple (NCT04285372)
Vascular Response of Orsiro vs. Xience Drug-Eluting Stents for Treating Coronary Bifurcation Lesions (NCT05200637)
Using 3D Imaging to Optimize Bifurcation Stenting (NCT02972489)
COmplex BifuRcation Lesions: a Comparison Between the AXXESS Device and Culotte Stenting: an Optical Coherence Tomography (OCT) Study (NCT01486095)
BiOSS LIM C vs 2nd Generation DES in Non-LM Bifurcations (NCT03548272)
Everolimus-Eluting Stent for Bifurcation Coronary Lesions: Comparison of Simple Versus Complex Techniques (NCT00916695)
Comparison of Results Achieved by Different Ballooning Techniques in Bifurcation Stenting (NCT05559424)
DCB vs. DES in Bifurcation Coronary Lesions (NCT06551662)
Side Branch Predilatation Strategy in Coronary Bifurcation Lesion Stenting (SBPS) (NCT01430377)
The Study of Active Transfer of Plaque Technique for Unprotected Distal Left Main Bifurcation Lesions (NCT02127138)
BIfurcation Optimal Treatment Strategy With LYMus Eluting Dedicated Bifurcation Versus Convention Stent Randomized Study (NCT01430364)
Double Rewire Versus Double Kissing Crush Stenting Technique in Left Main Bifurcation Lesions (NCT05258318)

The Bioresorbable Implants for Scaffolding Obstructions in Randomized Bifurcations (BIFSORB) Study (NCT02973529)
The COMPLETE Treatment of Bifurcation With Two-stent Techniques: Randomized Comparison of Crush Versus Culotte Technique (NCT05488665)
The European Bifurcation Club Randomized Trial of Stepwise Provisional Stenting Versus Drug Coated Balloon Therapy for Non-left Main True Coronary Bifurcations (NCT06822322)
Drug-coating Balloon Angioplasties for True Coronary Bifurcation Lesions (NCT04242134)
Comparison of PROVISIONal 1-stent Strategy with DEB Versus Planned 2-stent Strategy in Coronary Bifurcation Lesions (NCT06002932)
The Nordic Bifurcation Study III (NCT00914199)
Bingo Drug-eluting Balloon Versus a Drug-eluting Stent for Coronary Bifurcation Lesions (NCT06441539)
Comparison Between Everolimus-eluting Stent (EES) and Sirolimus-eluting Stent (SES) in the Bifurcation Lesion (NCT01266239)
Reverse T-stenting and Minimal Protrusion With External Minicrush for Treatment of Complex Coronary Bifurcation (NCT05782738)
The Study of Active Transfer of Plaque Technique for Non-Left Main Coronary Bifurcation Lesions (NCT02768116)
Simple Crossover Versus Side Branch Opening in Patients With Non-Left Main Bifurcation Lesion (NCT05705362)
Immediate and Short Term Outcomes for Using Drug Coated Balloons in Treating Coronary Bifurcation Lesions (NCT05872074)
A Study of Drug Coated Balloon For Treating the Side Branch in Complex Bifurcation Lesions (NCT05222061)
Jailed-balloon Technique in Coronary Bifurcation Lesion PCI (NCT02167230)
Sirolimus DEB in Coronary Bifurcation Lesions (NCT04896177)
Treatment of Bifurcated Coronary Lesions With Cypher™-Stent (NCT00288535)
Bifurcation PCI With a Hybrid Strategy With Drug Eluting Balloons Versus a Stepwise Provisional Two-stent Strategy (NCT05731687)
Yao Strategy for the Treatment of de Novo Medina 0,1,0 or 0,0,1 Bifurcation Lesion (NCT06166459)
The BIFSORB Pilot Study II (NCT03027856)
Trial of Orbital Atherectomy Versus Standard Strategy in Calcified Bifurcation Lesions (NCT06736899)

Drug Coated Balloon for Side Branch Treatment vs. Conventional Approach in True Bifurcation Coronary Disease: PRO-DAVID (NCT04403048)
Study of the Paclitaxel-Coated Balloon Catheter in Bifurcated Coronary Lesions (NCT01278186)
Conventional Versus Intentional StraTegy in Lesions With High Risk PrEdiction of Side Branch OccLusion in InterVEntion (NCT02644434)
Efficacy and Safety of Sirolimus-Coated Spiral Balloon for Coronary Bifurcation Lesions (NCT06618248)

Supplementary Table S2: Bifurcation-dedicated stents built as a main vessel stent with side branch access

Name & Company	Bifurcation Stent Design, Platform, Delivery	Approval Status
**Main branch stent with side branch access**		
Nile PAX stent (Minvasys, Genevilliers, France)	**Design**: Single stent with same metal:artery ratio loaded on two independent balloon catheters and wires; one balloon within main vessel stent and one balloon through the side branch port. Side branch port flared with kissing inflation. No jailed wires or wire-recrossing. Similar to Twin-Rail but two independent balloon catheters rather than single catheter<br>**Platform**: Cobalt-chromium 73um struts with paclitaxel coating 2.5 ug/mm2; balloon expandable<br>**Delivery**: Loaded on two independent monorail balloon catheters and wires	CE mark (2009) (1)
Twin Rail (Invatect S.r.l., Brescia, Italy)	**Design**: Stent premounted on double balloons in its proximal portion, and only on the MB balloon in its distal portion. Single dual lumen catheter splitting into two distal balloons with a central stopper that prevents further advancement of the SDS when the carina is reached. The stent is deployed by simultaneous kissing inflation with a single indeflator.<br>**Platform**: Stainless steel stent<br>**Delivery**: Loaded on two wires	CE mark (July 2010) (2)
Petal (Boston Scientific, Natick, MA, US)	**Design**: Single cylindrical stent with side branch "petal" struts that expands by inflating connected cylindrical and smaller elliptical balloons. Petal struts project 2mm into the side branch.<br>**Platform**: Paclitaxel-eluting platinum chromium alloy (more visibility than stainless steel or cobalt chromium and stronger)<br>**Delivery**: Loaded on two wires	N/A (3)
Multilink Frontier (Abbott Vascular Devices, CA/Guidant Corporation, Santa Clara, CA, USA)	**Design**: Y-shaped stent with a common inflation lumen and an integrated tip design that allows single tip delivery - MB balloon tip includes a pocket on the distal sleeve for joining the MB and SB balloon tips with a mandrel. The sleeve is withdrawn to separate the mandrel and unjoin the balloon tips. A 300cm wire is advanced into the OTW SB lumen and into the SB.<br>**Platform**: 18mm balloon expandable stainless steel stent<br>**Delivery**: Loaded on two wires (monorail for MB and OTW inner lumen for SB)	CE mark (2022)<br><br>FDA (2023) (4) (5)

Trieme (Trireme Medical Inc., CA, USA)	**Design**: Single stent with a SB support structure. SB stabilizing wire is encased in a peel-away lumen and advanced into SB when aligned.<br>**Platform**: Balloon expandable stainless steel stent<br>**Delivery**: Loaded on one wire	CE mark (2008) (6)
Side-kick (Y-med Inc, San Diego, CA, USA)	**Design**: MB fixed wire platform with rapid exchange steerable SB guide. Three models with different exit ports.<br>**Platform**: Bare metal and drug eluting stents<br>**Delivery**: Loaded on one wire	N/A (7)
BiOSS (Balton, Warsaw, Poland)	**Design**: Single stent with two parts with step-down in diameters, joined by two connecting struts spanning a 0.9-1.5 mm middle gap<br>**Platform**: 120 um stainless steel struts with polymer and paclitaxel or sirolimus<br>**Delivery**: Loaded on one wire	CE mark (2010) (8)
Stentys (Stentys S.A.S., Clichy, France)	**Design**: Single stent with Z-shaped interconnectors that break to allow scaffolding to side branch far wall<br>**Platform**: Nitinol 102um or 133 um struts with no, paclitaxel, or sirolimus coating; self-expanding<br>**Delivery**: Loaded on one wire	CE mark (2010) (9)

Supplementary Table S3: Bifurcation-dedicated stents built as a side branch stent with main vessel access; proximal main vessel stent; or pre-made bifurcation stent.

Name & Company	Bifurcation Stent Design, Platform, Delivery	Approval Status
**Side branch with main vessel access**		
Capella Side Guard (Capella Inc. MA, US)	**Design**: Flares proximally at ostium of side branch into a trumpet shape to achieve full ostial coverage. Innovative deployment with tearing of protective sheath. Addresses side branch underexpansion and inadequate SB stent coverage. No need for crushing and easier re-wiring due to single layer of stent after main vessel stenting<br>**Platform**: Self-expanding nitinol stent<br>**Delivery**: Loaded on one wire	CE mark (10)
Tryton (Tryton Medical, MA, US)	**Design**: Tri-ZONE technology with three segments with different strut patterns and density: Side Branch zone for side branch stenting; Transition Zone for side branch ostium coverage; and Main vessel zone has large opening for distal main vessel access. Different diameters in each segment.<br>**Platform**: Cobalt-chromium 84um struts; bare metal; balloon expandable<br>**Delivery**: Loaded on one wire	CE mark (2008)<br>FDA (2017) only device with specific left main indication (11) (12)
**Proximal main vessel**		
Axxess (Devax, Irvine, CA, US)	**Design**: Conically shaped stent for proximal main vessel. No metal at carina. Ideally suited for Medina 1,0,0.<br>**Platform**: Self-expanding 150 um strut nitinol biolimus eluting stent<br>**Delivery**: Loaded on wire towards most angulated or diseased branch	CE mark (July 2010)<br>UK market approval (13) (14)
**Bifurcated stent**		
Medtronic Bifurcated Stent (Medtronic, Santa Rosa, CA)	**Design**: Dual branch, Y-shaped stent with two balloons, one each in the main and side branch, inflated simultaneously through a single proximal port<br>**Platform**: Bare metal cobalt-based alloy stent with 91 um struts<br>**Delivery**: Advanced on two wires	Pre-market stage (15)
Advanced Bifurcation System (ABS, Los Angeles, CA, USA)	**Design**: Independently movable, mother and daughter balloon catheters. Daughter catheter slides through a port on the mother catheter<br>**Platform**: 105 um stainless steel struts, uncoated<br>**Delivery**: Advanced on two wires	Pre-market stage(16)

Supplemental References

1. Berland J. The Nile CroCo and Nile PAX stents. EuroIntervention. 2015;11 Suppl V:V149-150.

2. Capodanno D, Dipasqua F, Tamburino C. Novel drug-eluting stents in the treatment of de novo coronary lesions. Vasc Health Risk Manag. 2011;7:103–18.

3. Ormiston JA, De Vroey F, Webster MWI, Kandzari DE. The Petal dedicated bifurcation stent. EuroIntervention. 2010 Dec;6 Suppl J:J139-142.

4. Lauren Mueller Doran. Medtronic launches latest generation drug-eluting coronary stent system following CE Mark approval [Internet]. 2022. Available from: https://news.medtronic.com/Medtronic-launches-latest-generation-drug-eluting-coronary-stent-system-following-CE-Mark-approval

5. Premarket Approval (PMA) [Internet]. 2023. Resolute Onyx Zotarolimus-Eluting Coronary Stent System, Onyx Frontier Zotarolimus-Eluting Coronary Stent System. Available from: https://www.accessdata.fda.gov/scripts/cdrh/cfdocs/cfpma/pma.cfm?id=P160043S058

6. TriReme Medical Inc. Receives CE Marking for Antares(TM) Coronary Stent System [Internet]. 2008. Available from: https://www.biospace.com/trireme-medical-inc-receives-ce-marking-for-antares-tm-coronary-stent-system

7. Shao-Liang C, Nanjing First Hospital, Nanjing Medical University, Nanjing, Jiangsu, China. E: chmengx@126.com, Sheiban I, Interventional Cardiology, Division of Cardiology, University of Turin. Dedicated Bifurcation Stents Strategy. Interventional Cardiology Review. 2009;4(1):70.

8. Gil RJ, Bil J, Kern A, Pawłowski T. First-in-man study of dedicated bifurcation cobalt-chromium sirolimus-eluting stent BiOSS LIM C® - three-month results. Kardiol Pol. 2018;76(2):464–70.

9. Pyxaras SA, Schmitz T, Naber CK. The STENTYS Self-Apposing® stent. EuroIntervention. 2015 May;11(V):V147–8.

10. David Blossom. Cappella Medical Devices Ltd Announces Excellent Long Term Follow-Up Clinical Data on its Sideguard(R) Technology [Internet]. 2011. Available from: https://www.biospace.com/cappella-medical-devices-ltd-announces-excellent-long-term-follow-up-clinical-data-on-its-sideguard-r-technology

11. Tryton Medical, Inc. Receives CE Mark Approval for its Side-Branch Stent [Internet]. 2008. Available from: https://www.biospace.com/tryton-medical-inc-receives-ce-mark-approval-for-its-side-branch-stent

12. Premarket Approval (PMA) [Internet]. 2017. TRYTON SIDE BRANCH STENT. Available from: https://www.accessdata.fda.gov/scripts/cdrh/cfdocs/cfpma/pma.cfm?ID=320643

13. Rawlins J, Din J, Talwar S, O'Kane P. AXXESS™ Stent: Delivery Indications and Outcomes. Interv Cardiol. 2015 May;10(2):85–9.

14. Jeff Thiel. Devax, Inc. Receives CE Mark for the AXXESS Drug Eluting Bifurcation Stent [Internet]. 2010. Available from: https://www.biospace.com/devax-inc-receives-ce-mark-for-the-axxess-drug-eluting-bifurcation-stent

15. Meredith I, Worthley S, Whitbourn R, Webster M, Fitzgerald P, Ormiston J. First-in-human experience with the Medtronic Bifurcation Stent System. EuroIntervention. 2011 Oct;7(6):662–9.

16. Khorsandi M, Abizaid A, Dani S, Bourang H, Costa RA, Kar S, et al. The ABS mother-daughter platforms. EuroIntervention. 2015 May;11(V):V151–2.
